# Novel Hendra Virus Variant Circulating in Black Flying Foxes and Grey-Headed Flying Foxes, Australia

**DOI:** 10.3201/eid2805.212338

**Published:** 2022-05

**Authors:** Alison J. Peel, Claude Kwe Yinda, Edward J. Annand, Adrienne S. Dale, Peggy Eby, John-Sebastian Eden, Devin N. Jones, Maureen K. Kessler, Tamika J. Lunn, Tim Pearson, Jonathan E. Schulz, Ina L. Smith, Vincent J. Munster, Raina K. Plowright, Bat One Health Group

**Affiliations:** Griffith University Centre for Planetary Health and Food Security, Nathan, Queensland, Australia (A.J. Peel, P. Eby, T.J. Lunn);; National Institutes of Health, Hamilton, Montana, USA (C.K. Yinda, J.E. Schulz, V.J. Munster);; EquiEpiVet, Aireys Inlet, Victoria, Australia (E.J. Annand);; Department of Agriculture, Water, and the Environment, Canberra, Australian Capital Territory, Australia (E.J. Annand);; University of Sydney, Sydney, New South Wales, Australia (E.J. Annand, J.-S. Eden);; Texas Tech University, Lubbock, Texas, USA (A.S. Dale); University of New South Wales, Sydney (P. Eby);; Montana State University, Bozeman, Montana, USA (D.N. Jones, M.K. Kessler, R.K. Plowright);; Bellingen, New South Wales, Australia (T. Pearson);; CSIRO, Black Mountain, Australian Capital Territory, Australia (I.L. Smith)

**Keywords:** Hendra virus, henipavirus, viruses, reservoir host, Pteropus, flying foxes, bats, emerging infections, zoonoses

## Abstract

A novel Hendra virus variant, genotype 2, was recently discovered in a horse that died after acute illness and in *Pteropus* flying fox tissues in Australia. We detected the variant in flying fox urine, the pathway relevant for spillover, supporting an expanded geographic range of Hendra virus risk to horses and humans.

Hendra virus (HeV; genus *Henipavirus*, family *Paramyxoviridae*) is a well-characterised zoonotic pathogen endemic to *Pteropus* spp. bats (flying foxes) in Australia. Spillover from bats to horses has been detected 63 times; 4 of 7 persons infected from horses have died (*1*). Quantitative reverse-transcription PCR (qRT-PCR) (*2*) is a tool used for surveillance and priority disease investigation in bats and horses (*3*,*4*). The high specificity of assays limits detection to a narrow range of genotypic diversity, meaning that divergent variants might remain undetected (*3*). 

In October 2021, spillover of a novel variant, HeV genotype 2 (HeV-g2), resulted in the death of a horse in New South Wales (NSW), Australia, farther south than HeV had previously been detected in horses (*5*). This spillover was detected only because diagnostic assays had been recently updated after retrospective discovery of HeV-g2 in a horse that exhibited signs of HeV disease in 2015 but tested negative through routine screening at that time (*3*). Discovery of HeV-g2 in this horse arose using broad panparamyxovirus PCRs (*6*), followed by next-generation sequencing and virus isolation. The variant showed 84% pairwise nucleotide identity genomewide to prototype HeV (HeV-g1), and 99% similarity with partial sequences recovered from tissue samples from a grey-headed flying fox, *P. poliocephalus* (*7*). Bats submitted for lyssavirus diagnostics were opportunistically screened using an updated quantitative PCR specific for HeV-g2, which resulted in additional positive detections in tissue collected from *P. poliocephalus* in 2019–2021 and a little red flying fox (*P. scapulatus*) in 2015 (*7*).

Although HeV-g1 has been detected in tissues from all 4 flying fox species in continental Australia, excretion of the virus has been confirmed only in the black flying fox (*P. alecto*) and the spectacled flying fox (*P. conspicillatus*), suggesting these species are sources of transmission to horses (*8*,*9*). Sequence mismatches between HeV-g1 and HeV-g2 mean that PCR assays used in previous surveillance of reservoir hosts would not have detected the novel HeV-g2. To address this gap, we used a new qRT-PCR (*3*) to screen banked flying fox urine samples collected over a large extent of space and time. 

## The Study

We collected pooled urine samples from plastic sheets placed underneath flying fox roosts in southeastern Queensland and mid- to north-coast NSW during December 2016–September 2020 (Figure). We placed sheets in areas of the roost where *P. alecto* flying foxes were roosting, although other species were often also present. We recorded the number and species of bats immediately above the sheets. We also captured individual bats in mist nests; recorded species, sex, and age class; then collected urine samples directly from each anaesthetised bat or from a urine collection bag attached to its holding bag. Shortly after collection, we placed samples into viral lysis buffer, virus transport media, or an empty cryovial and stored them at −80°C (Appendix). 

**Figure:**
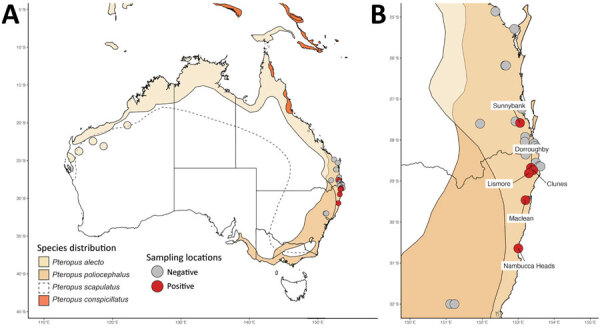
Distribution of flying fox species in Australia (*13*) and sampling locations for study of HeV variant circulating in flying foxes in southeastern Queensland and mid- to north-coast New South Wales, December 2016–September 2020. A) Locations in Australia; B) locations in study area. HeV, Hendra virus; HeV-g2, HeV genotype 2.

We used the QIAamp Viral RNA Kit using a QIAcube HT automated system (QIAGEN, https://www.qiagen.com) to extract RNA, then eluted it in 150 μL of TE buffer and first screened it for HeV-g1 using a qRT-PCR assay targeting the P gene (Table 1). We stored extracted RNA at −80°C and then screened it for HeV-g2 using the new multiplexed qRT-PCR assay, targeting the M gene with primers specific for HeV-g1 and HeV-g2 (*2*,*3*) (Table 1; Appendix). We used 10-fold dilutions with a known number of genome copies to construct a standard curve, calculate copy numbers/mL, and estimate limit of detection. We amplified the partial cytochrome *b* gene from all positive samples (*10*,*11*) (Table 1) and confirmed host species identity based on sequence identity across 402-bp sequences (Appendix). 

**Table 1:** Primers and probes used in PCR for study of novel Hendra virus variant circulating in black and grey-headed flying foxes, Australia*

Target	Primers and Probes	Reference
HeV-g1 P gene	F: 5′-CCCAACCAAGAAAGCAAGAG	This study
	R: 5′-TTCATTCCTCGTGACAGCAC	
	P: 5′-TTACTGCGGAGAATGTCCAACTGAGTG	
HeV-g1 M gene	F: 5′-CTTCGACAAAGACGGAACCAA	(*2*)
	R: 5′ TGGCATCTTTCATGCTCCATCTCGG	
	P: 5′ CCAGCTCGTCGGACAAAATT	
HeV-g2 M gene	F: 5′ TCTCGACAAGGACGGAGCTAA	(*3*)
	R: 5′ CCGGCTCGTCGAACAAAATT	
	P: 5′ TGGCATCCTTCATGCTTCACCTTGG	
Partial cytochrome b gene	F: 5′-CGAAGCTTGATATGAAAAACCATCGTTG	(*10*,*11*)
	R: 5′ AACTGCAGCCCCTCAGAATGATATTTGTCCTCA	
*F, forward; R, reverse; P, probe.		

We screened 4,539 pooled urine samples collected from 129 underroost sampling sessions and 1,674 urine samples collected from individual bats over 39 catching sessions during July 2017–September 2020 (Appendix Tables 1, 2). Eight pooled urine samples and 2 samples from individual flying foxes tested positive for HeV-g2 (Table 2). Positive samples were from Sunnybank in Queensland and Clunes, Lismore, Dorroughby, Maclean, and Nambucca Heads in NSW.

**Table 2:** Details of urine samples collected from *Pteropus alecto* and *P. poliocephalus* flying foxes in underroost sampling sessions that tested positive for HeV-g2 and associated session-level prevalence for HeV-g1 and HeV-g2, Australia*

Site	Date	HeV-g2			HeV-g1		Sample ID	RNA copies/ mL†	Species recorded‡	*Cyt b* species§
		No. positive/ total	Prevalence, % (95% CI)		No. positive/ total	Prevalence, % (95% CI)				
Clunes, NSW	2019 Jul 27	1/36	2.8 (0.1–16.2)		0/36	0.0 (0–12.0)	ACMAC001_35_1	169	*Pa*	*Pa*
Maclean, NSW	2018 Jul 9	1/36	2.8 (0.1–16.2)		0/36	0.0 (0–12.0)	ACCLU004_22_1F	225	*Pp*	*Pp*
Clunes, NSW	2017 Aug 8	1/36	2.8 (0.1–16.2)		5/36	13.9 (5.2–30.3)	ACMAC001_35_1	174	2 *Pa*; 0 *Pp*	*Pa*
Clunes, NSW	2018 Nov 1	2/51	3.9 (0.7–14.6)		4/51	7.8 (2.5–19.7)	ARCLU002_14_1	38	0 *Pa*; 2 *Pp*	Mixed *Pp/Pa*
							ARCLU010_22_1	17	1 *Pa*; 2 *Pp*	*Pa*
Lismore, NSW	2017 Aug 27	1/48	2.1 (0.1–12.5)		21/48	43.8 (29.8–58.7)	ARCLU010_26_1	783	4 *Pa*; 0 *Pp*	NA
Nambucca Heads, NSW	2018 May 20	2/31	6.5 (1.1–22.8)		8/31	25.8 (12.5–50.1)	ARLIS002_55_1	67	0 *Pa*; 2 *Pp*	*Pa*
							ARNAM005_2_1	15	4 *Pa*; 0 *Pp*	*Pa*
Sunnybank, QLD	2018 Nov 26	1/36	2.8 (0.1–16.2)		1/36	2.8 (0.1–16.2)	ARNAM005_12_1	381,123	0 *Pa*; 4 *Pp*	*Pp*
Dorroughby, NSW	2016 Dec 16	1/18	2.5 (0.01–14.7)		1/18	2.5 (0.01–14.7)	ARSUN015_15_1	58	NR	*Pa*

*Cyt b, Cytochrome b; HeV, Hendra virus; NSW, New South Wales; *Pa*, *P. alecto*; *Pp, P. poliocephalus*; QLD, Queensland; NA, not available; NR, not recorded. †HeV-g2 viral copies/mL: the minimum copy number which would be expected to reliably give a positive PCR result in all replicates in the quantitative reverse transcription PCR assay (the limit of detection) was 5–10 copies per reaction (≈1,070–2,140 copies/mL). ‡For underroost samples, the number of flying foxes recorded by species (*P. alecto* or *P. poliocephalus*) at the time of sampling might not precisely reflect the proportion of urine collected from each species. §Appendix Table 3 (https://wwwnc.cdc.gov/EID/article/28/5/21-2338-App1.pdf).

We detected HeV-g2 in samples collected across all seasons. Prevalence in sessions with positive detections ranged from 2.5% to 6.5% (95% CI 0.1%–22.8%). In pooled samples, HeV-g2 was only detected in sessions when HeV-g1 was also detected (HeV-g1 prevalence range 2.5%–50.1%); however, we found no statistically significant correlation between HeV-g1 and HeV-g2 prevalence (Pearson correlation analysis ρ = 0.09; p = 0.87). Most (8/10) of the HeV-g2–positive samples had low genome copies, but 2, ARSUN015_15_1 and ARLIS002_55_1, had considerably higher copy numbers (Table 2). 

Individual flying foxes that tested positive included a *P. poliocephalus* juvenile female captured in Maclean, NSW, and a *P. alecto* adult male captured in Clunes, NSW (Appendix Table 3). We detected HeV-g2 in pooled samples from mixed-species roosts containing *P. alecto* and *P. poliocephalus* flying foxes. Cytochrome b sequencing identified DNA from *P. alecto* flying foxes in 6/8 positive underroost samples and from *P. poliocephalus* flying foxes in 2/8 (Table 2). 

## Conclusions

Urine is the route of HeV excretion from flying foxes and the source of virus transmission to horses. Detecting the novel Hendra variant HeV-g2 in the urine of flying foxes helped identify its distribution range, associated host species, transmission dynamics, and spillover risk. We show evidence that *P. alecto* and *P. poliocephalus* flying foxes excrete HeV-g2 in urine and both are likely competent reservoir hosts. We did not screen urine samples from *P. conspicillatus* or *P. scapulatus* flying foxes, so the potential of these species to excrete HeV-g2 in urine remains unconfirmed. 

Although HeV-g1 has been detected in flying fox urine samples collected across all seasons, prevalence peaks in winter in subtropical regions (*4*,*12*), which is consistent with our preliminary HeV-g2 seasonality findings (5/8 detections in late May–late August) in the study area. The significantly lower prevalence of HeV-g2 than HeV-g1 could indicate actual lower prevalence in the sampled population. Alternatively, repeated freeze-thaw cycles in our samples or the bias toward collecting *P. alecto* urine in our sampling design might have led to lower detection. Tissue samples from flying foxes submitted for lyssavirus testing after contact with humans or pets showed higher HeV-g2 prevalence than our samples from wild populations (*7*), which might reflect higher prevalence in sick or stressed bats or geographical differences. HeV-g2 was previously detected in tissue samples from South Australia (3 positives from 4 samples), Victoria (7/64), and Western Australia (1/2) (*7*). Our findings extend the known distributional range of HeV-g2 to southeastern Queensland and mid- to north-coast NSW, areas proximate to the 2 known cases of HeV-g2 spillover to horses (*3*,*5*).

Our findings support expanding the expected geographic risk area for HeV spillover to include the distribution of *P. poliocephalus* flying foxes. Screening flying fox urine samples from a broader geographic range, including regions where *P. alecto* flying foxes are absent, should better inform epidemiologic relationships and relative prevalence of HeV variants. Given that data on the true diversity of HeV and related viruses in flying fox populations are incomplete, unbiased or *Paramyxoviridae* family–level viral surveillance in reservoir and spillover hosts might identify further variants. Developing a panel of diagnostic tools to detect a more comprehensive range of the viruses capable of spillover would substantially advance our ability to forecast spillover risk, manage biosecurity, and provide guidance to horse owners, veterinarians, and other stakeholders.

Appendix Additional information on novel Hendra virus variant circulating in black flying foxes and grey-headed flying foxes, Australia.
