# Supplementary material for: Novel Hendra Virus Variant Circulating in Black Flying Foxes and Grey-Headed Flying Foxes, Australia
Source: Emerg Infect Dis. 2022 May;28(5):1043–7. doi: 10.3201/eid2805.212338 (PMC9045453; doi:10.3201/eid2805.212338)
Supplement: Appendix — Additional information on novel Hendra virus variant circulating in black flying foxes and grey-headed flying foxes, Australia. [file 21-2338-Techapp-s1.pdf]

Article DOI: https://doi.org/10.3201/eid2805.212338

Novel Hendra Virus Variant Circulating in Black Flying Foxes and Grey-Headed Flying Foxes

Appendix

METHODS

Sample Collection

Sample collection was covered under Griffith University Animal Ethics Committee Approval ENV 10 16 AEC.

Under-Roost Urine samples

Urine samples were collected from plastic sheets placed underneath flying fox roosts in southeast Queensland and mid- to north-coast New South Wales during December 2016–September 2020 (Figure 1). In each sampling session, up to 64 plastic sheets (0.9 m×1.3 m) were placed under roosting trees before dawn as decribed elsewhere (*1*). After bats had returned to the roost, a single pooled urine sample from each sheet was pipetted into a tube with AVL lysis buffer (QIAGEN; https://www.qiagen.com); the target amounts were 140 µL of urine into 560 µL buffer, 200–1000 µL of urine into 1000 µL of viral transport medium, or a plain cryovial. Sample collection occurred within approximately 7 hours of laying sheets (<6 h of sunrise). Samples were transferred to a CryoShipper (below –80°C) for transport and stored at –80°C in the laboratory. Species present within the roost at the time of sampling included black flying foxes (*Pteropus alecto*), grey-headed flying foxes (*P. poliocephalus*), and more rarely, little red flying foxes (*P. scapulatus*) (*2*). Where possible, sheet placements within the roosting area were prioritized towards where *P. alecto* and away from where *P. scapulatus* were roosting. The number and species of bats immediately above the sheet was recorded; however, in some cases individual bats were easily disturbed and took flight, meaning that these data are indicative only.

Samples from Captured Individuals

Urine samples were also collected directly from individual bats captured in mist nests at their roost site, using methods described elsewhere (Hansen et al., in review). Bats were held in cotton bags with the bottom third lined with plastic and a urine collection bag attached to facilitate the collection of samples. Bats were anaesthetised for further sample collection and their species, sex, and age class (adult, subadult, juvenile) were recorded. Urine samples were collected directly from the bat if it urinated while under anaesthetic, or from the urine collection bag. The former were prioritised for screening if both were available. Urine samples were placed into AVL buffer, viral transport medium or a plain cryovial and stored as described above.

Sample Selection

Over 10,000 urine samples were collected in combined under-roost and individual capture sessions. We selected a subset for Hendra virus genotype 2 (HeV-g2) testing using criteria that address the 2 main aims of the work: describing the distribution and dynamics of HeV-g2 in southeast Queensland and northeast New South Wales and exploring host species associations. Higher rates of detection were observed in under-roost sampling, as multiple individual bats above a sheet might contribute to each pooled sample (*3*). Screening samples collected using this approach maximizes the likelihood of detecting novel HeV-g2 variant if it is present. To explore distribution and dynamics, we screened 4,322 pooled urine samples collected from 127 under-roost sampling sessions during July 2017–September 2020. Samples were selected to represent the broad spatiotemporal coverage of the sample set (Appendix Table 1). To address host species associations, we selected samples attributed to either *P. alecto* or *P. poliocephalus*. Initially, we screened all available samples collected from individual bats where the species was identified in the field (674 urine samples collected from individual bats over 39 catching sessions during August 2017–September 2020, Appendix Table 2). Because the number of samples from captured bats was biased towards *P. alecto*, we included an additional 217 under-roost samples from 2 sessions with high proportions of *P. poliocephalus* (Maclean and Stewarts Brook, Appendix Table 1).

***Cytochrome b* Sequencing for Species Identification**

Partial *cytochrome b* gene was amplified by PCR from all positive samples using previously described primers validated for species identification (Table 1). For amplification of *cytochrome b* DNA from each sample, 2 μL of complementary DNA template and primers at 0.2

μM each were used in a 25 μL reaction with the TopTaq master mix PCR premix (QIAGEN) and amplified with 35 cycles at 94°C for 30 s, 50°C for 30 s, and 72°C for 1.5 min. PCR products were run on 1% agarose gel and positive bands were excised and purified using NucleoSpin Gel and PCR Clean-up kit according to manufacturer instructions (MACHEREY-NAGE; https://www.mn-net.com). PCR products were Sanger sequenced (ACGT) and species confirmed based on >98% sequence identity across 402 bp length sequences. *Cytochrome b* sequences are listed in Appendix Table 3 below.

References

1. Edson D, Field H, McMichael L, Vidgen M, Goldspink L, Broos A, et al. Routes of Hendra virus excretion in naturally-infected flying-foxes: implications for viral transmission and spillover risk. PLoS One. 2015;10:e0140670. PubMed https://doi.org/10.1371/journal.pone.0140670

2. Lunn TJ, Eby P, Brooks R, McCallum H, Plowright RK, Kessler MK, et al. Conventional wisdom on roosting behavior of Australian flying-foxes-A critical review, and evaluation using new data. Ecol Evol. 2021;11:13532–58. PubMed https://doi.org/10.1002/ece3.8079

3. Giles JR, Peel AJ, Wells K, Plowright RK, McCallum H, Restif O. Optimizing noninvasive sampling of a zoonotic bat virus. Ecol Evol. 2021;11:12307–21. PubMed https://doi.org/10.1002/ece3.7830

4. Neaves LE, Danks M, Lott MJ, Dennison S, Frankham GJ, King A, et al. Unmasking the complexity of species identification in Australasian flying-foxes. PLoS One. 2018;13:e0194908. PubMed https://doi.org/10.1371/journal.pone.0194908

5. Tsang SM, Wiantoro S, Veluz MJ, Sugita N, Nguyen YL, Simmons NB, et al. Dispersal out of Wallacea spurs diversification of *Pteropus* flying foxes, the world's largest bats (Mammalia: Chiroptera). J Biogeogr. 2020;47:527–37. PubMed https://doi.org/10.1111/jbi.13750

Appendix Table 1. Detections of HeV-g2 by under-roost sampling sessions

Site	Start date	Positive	Total
Anchorage	2017 Aug 6	0	22
Anchorage	2017 Aug 31	0	26
Banora Green	2017 Aug 30	0	28
Burleigh	2018 Jul 1	0	36
Burleigh	2018 Jul 31	0	5
Burleigh	2018 Aug 31	0	9
Burleigh	2018 Nov 24	0	8
Burleigh	2019 Jan 3	0	32
Burleigh	2019 Jan 23	0	4
Burleigh	2019 Mar 6	0	4
Burleigh	2019 Mar 23	0	3
Burleigh	2019 Jul 26	0	3
Burleigh	2019 Oct 30	0	39
Burleigh	2020 Aug 24	0	9
Burleigh	2020 Sep 29	0	27
Byron Bay	2018 Jun 17	0	34
Canungra	2017 Aug 28	0	45
Canungra	2017 Sep 30	0	28
Canungra	2017 Oct 28	0	45
Canungra	2018 Jan 20	0	1
Clunes	2017 Jul 17	0	30
Clunes	2017 Aug 8	1	36
Clunes	2017 Sep 4	0	47
Clunes	2018 Feb 14	0	35
Clunes	2018 May 14	0	50
Clunes	2018 Jun 11	0	43
Clunes	2018 Aug 1	0	45
Clunes	2018 Aug 30	0	54
Clunes	2018 Oct 10	0	37
Clunes	2018 Nov 1	2	51
Clunes	2018 Nov 29	0	33
Clunes	2019 Jan 5	0	43
Clunes	2019 Jan 26	0	29
Clunes	2019 Feb 27	0	38
Clunes	2019 Mar 22	0	57
Clunes	2019 May 7	0	62
Clunes	2019 Jun 5	0	50
Clunes	2019 Jul 2	0	64
Clunes	2019 Jul 24	0	64
Clunes	2019 Aug 31	0	39
Clunes	2019 Sep 24	0	49
Clunes	2019 Oct 24	0	55
Clunes	2019 Nov 27	0	49
Clunes	2019 Dec 19	0	50
Clunes	2020 Jan 23	0	48
Clunes	2020 Feb 20	0	51
Clunes	2020 Mar 25	0	55
Clunes	2020 May 1	0	47
Clunes	2020 May 28	0	59
Clunes	2020 Jun 26	0	60
Clunes	2020 Aug 6	0	43
Clunes	2020 Aug 28	0	20
Clunes	2020 Sep 30	0	35
Commissioner’s Gully	2020 Aug 25	0	7
Currumbin	2018 Oct 4	0	50
Dorroughby	2016 Dec 16	1	18
Gympie	2019 Jan 30	0	39
Hervey Bay	2018 Jul 23	0	26
Hervey Bay	2020 Jul 27	0	12
Lismore	2016 Dec 18	0	14
Lismore	2017 Aug 27	1	48
Lismore	2017 Oct 1	0	42
Lismore	2017 Oct 29	0	33
Lismore	2017 Nov 25	0	29
Lismore	2017 Dec 17	0	31
Lismore	2018 Sep 16	0	14
Lismore	2020 Aug 29	0	35
Maclean	2018 Jul 7	0	51

Site	Start date	Positive	Total
Mount Ommaney	2019 Jan 15	0	39
Mullumbimby	2017 Sep 5	0	37
Nambucca Heads	2017 Jul 26	0	28
Nambucca Heads	2017 Sep 2	0	35
Nambucca Heads	2017 Dec 17	0	15
Nambucca Heads	2018 Feb 11	0	18
Nambucca Heads	2018 May 20	2	31
Nambucca Heads	2018 Jul 22	0	17
Nambucca Heads	2018 Sep 2	0	16
Nambucca Heads	2018 Nov 25	0	8
Nambucca Heads	2019 Feb 17	0	15
Nambucca Heads	2019 Apr 28	0	20
Nambucca Heads	2019 Aug 13	0	37
Redcliffe	2018 Apr 27	0	30
Redcliffe	2018 May 24	0	51
Redcliffe	2018 Jun 29	0	56
Redcliffe	2018 Jul 26	0	57
Redcliffe	2018 Aug 28	0	43
Redcliffe	2018 Oct 3	0	32
Redcliffe	2018 Oct 31	0	33
Redcliffe	2018 Nov 30	0	36
Redcliffe	2019 Jan 4	0	27
Redcliffe	2019 Feb 14	0	30
Redcliffe	2019 Feb 28	0	23
Redcliffe	2019 Mar 25	0	43
Redcliffe	2019 Apr 29	0	35
Redcliffe	2019 Jul 9	0	41
Redcliffe	2019 Aug 2	0	26
Redcliffe	2019 Aug 30	0	47
Redcliffe	2019 Sep 27	0	50
Redcliffe	2019 Oct 25	0	35
Redcliffe	2019 Nov 29	0	54
Redcliffe	2019 Dec 20	0	1
Redcliffe	2020 Jan 24	0	38
Redcliffe	2020 Feb 26	0	52
Redcliffe	2020 Apr 3	0	36
Redcliffe	2020 Apr 24	0	31
Redcliffe	2020 May 22	0	39
Redcliffe	2020 Jun 24	0	33
Redcliffe	2020 Aug 5	0	51
Redcliffe	2020 Aug 26	0	52
Redcliffe	2020 Sep 23	0	1
Scone	2019 Jun 17	0	22
Stewarts Brook	2019 Jun 19	0	166
Simpson’s Creek	2017 Aug 23	0	49
Stokers Siding	2020 Jun 5	0	37
Stokers Siding	2020 Aug 1	0	26
Sunnybank	2017 Nov 23	0	7
Sunnybank	2018 Jan 22	0	4
Sunnybank	2018 Mar 1	0	2
Sunnybank	2018 Mar 13	0	1
Sunnybank	2018 Oct 27	0	11
Sunnybank	2018 Nov 26	1	36
Sunnybank	2019 Jan 2	0	52
Sunnybank	2020 Jan 29	0	27
Sunnybank	2020 Sep 25	0	33
Toowoomba	2018 Nov 27	0	48
Toowoomba	2018 Dec 23	0	46
Toowoomba	2019 May 2	0	46
Toowoomba	2019 Oct 29	0	13
Tyalgum	2019 Feb 9	0	48

Appendix Table 2. Detections of HeV-g2 in individual bats by capture sessions

Site	Start date	Positive	Total
Bundaberg	2020 Feb 5	0	15
Clunes	2017 Aug 12	0	17
Clunes	2018 Feb 19	0	1
Clunes	2018 Aug 2	0	13
Clunes	2019 Jul 25	1	36
Gympie	2019 Jan 31	0	32
Hervey Bay	2018 Jul 15	0	40
Hervey Bay	2020 Jul 28	0	59
Maclean	2018 Jul 9	1	36
Mount Ommaney	2019 Jan 17	0	42
Redcliffe	2018 May 25	0	47
Redcliffe	2018 Jul 27	0	51
Redcliffe	2018 Sep 14	0	54
Redcliffe	2018 Dec 14	0	49
Redcliffe	2019 Mar 8	0	47
Redcliffe	2019 May 28	0	31
Redcliffe	2019 Jul 9	0	50
Redcliffe	2019 Sep 10	0	49
Redcliffe	2019 Dec 3	0	51
Redcliffe	2020 Mar 3	0	48
Redcliffe	2020 May 11	0	63
Redcliffe	2020 Jul 7	0	53
Redcliffe	2020 Sep 7	0	52
Sunnybank	2018 Mar 16	0	36
Toowoomba	2018 Jun 3	0	52
Toowoomba	2018 Jul 21	0	52
Toowoomba	2018 Sep 8	0	55
Toowoomba	2018 Dec 8	0	61
Toowoomba	2019 Jan 11	0	8
Toowoomba	2019 Mar 15	0	49
Toowoomba	2019 May 14	0	54
Toowoomba	2019 Jul 2	0	56
Toowoomba	2019 Jul 23	0	4
Toowoomba	2019 Sep 3	0	57
Toowoomba	2019 Dec 10	0	48
Toowoomba	2020 Mar 10	0	54
Toowoomba	2020 May 4	0	54
Toowoomba	2020 Jul 14	0	47
Toowoomba	2020 Sep 1	0	46

Appendix Table 3. Cytochrome b sequences and species assessment for each sample

Sample ID	Cytochrome b sequence	HQ, %*	Assessment
ACCLU004_22_1F	TATTTCAACTACAAGAACCACAATGACAAACATCCGTAAATCACACCCAC<br>TATTCAAAATTATCAACGACTCACTGATCGACCTACCCGCCCCATCAAGT<br>ATTTCCTCATGATGAAACTTCGGCTCACTACTAGGCATCTGTCTAGCCAT<br>CCAGATCCTAACAGGACTGTTCCTAGCTATACACTACACTTCAGACACAA<br>CGACCGCCTTCCAATCCGTAACTCATATCTGCCGAGACGTAAACTACGGA<br>TGAATCCTGCGTTATTTGCATGCTAACGGAGCATCCATATTCTTCATCTG<br>CCTATTCTTACATGTAGGCCGAGGCCTCTACTACGGATCTTACATTTACA<br>AAGAAACCTGAAACGTAGGTGTTATTCTCCTATTTGCTGTAATAGCAACA<br>GCC	85.4	*Pteropus alecto*<br>Cytb sequence has 100% match to *P. griseus* (KJ532423.1) and 98% match to *P alecto* (MN511367.1). This bat was captured in NSW and identified as *P. alecto* in the field. *P. griseus* is found in Indonesia but there is growing evidence from multiple studies (*4*,*5*) that *P. alecto* is poorly resolved relative to other species, and Australian populations include a newly recognised mitochondrial DNA lineage that suggests historical admixture with *P. griseus* (*4*). This individual was therefore assessed as *P. alecto*.
ACMAC001_35_1	TACTTCAACTACAAGAACCACAATGACAAACATCCGAAAATCACACCCAC<br>TATTCAAAATTATCAACGACTCACTAGTCGACCTGCCCGCTCCATCAAGT<br>ATCTCATCATGATGAAACTTTGGCTCACTACTAGGCATCTGCCTAGCCAT<br>CCAAATCCTGACAGGACTGTTCCTAGCCATACACTACACTTCAGACACAA<br>CAACCGCCTTCCAATCCGTGACTCACATCTGCCGAGACGTAAACTACGGA<br>TGAATCCTCCGCTACTTACACGCTAACGGAGCATCCATATTCTTCATCTG<br>CCTATTCCTACATGTGGGCCGAGGCCTCTACTACGGATCTTACATCTATA<br>AAGAGACCTGAAACGTAGGTGTCATCCTTCTATTTGCCGTAATAGCAACA<br>GCC	94.7	*P. poliocephalus*<br>Cytb sequence has 99.48% match to *P. poliocephalus* (KJ532404.1). This bat was captured in NSW and identified as *P. poliocephalus* in the field.
ARCLU002_14_1	TACTTCAACTACAAGAACCACAATGACAAACATCCGCAAATCACACCCAC<br>TATTCAAAGTTATCAACGACTCACTGATCGACCTACCCGCCCCATCAAGT<br>ATTTCCTCATGATGAAACTTCGGCTCACTACTAGGCATCTGCCTAGCCAT<br>CCAAATCCTAACAGGACTGTTCCTAGCTATACACTACACTTCAGACACAA<br>CGACCGCCTTCCAATCCGTGACCCATATCTGCCGAGACGTAAACTACGGA<br>TGAATTCTGCGTTATTTACATGCTAACGGAGCATCCATATTCTTCATCTG<br>CCTATTCTTACATGTAGGCCGAGGCCTCTACTACGGATCTTATATCTACA<br>AAGAAACCTGAAACGTAGGTGTTATTCTCCTATTTGCCGTAATAGCAACA<br>GCC	94.6	*P. alecto*<br>Cytb sequence has 99.48% match to *P. alecto* (KF726143.1). Two *P. alecto* were noted as roosting above the plastic sheet at the time of collection. No mixed peaks observed in chromatogram.
ARCLU010_22_1	TATTTCAACTACAAGAACCACAATGACAACCATCCGAAAATCACACCCCC<br>TATTCAAAATAATCAACCACTCATTAGTCGACCTACCCGCTCCATCAAGT<br>ATCTCATCATGATGAAACTTTGGCTCACTCCTAGGCATCTGCCTAACCAT<br>CCAAATCACCACAGGACTGTTCCTAGCCATACACTACACTTCAGACACAT<br>CAACCGCCTTTCAATCAATGACTCACATCAGCCGAGACGTAAATTACGGA<br>TGAATCATCCGCTACTTACACGCCAACGGAGCATCCATATTCTTCATCTG<br>CCTATTCCTACACATGGGCCGAGGCCTATATTACGGATCTTACATCTATA<br>AAGAGACCTGAAACAGTAGCATCATCCTCCTATTTGCAGTAATAGCAACA<br>GCC	33.0	Mixed *P. poliocephalus* and *P alecto*<br>Cytb sequence has 92.67% match to *P. poliocephalus* (FJ561387.1), however it is a low-quality sequence due to multiple mixed peaks, where the alternative peak is consistent with *P. alecto* sequences. Two *P. poliocephalus* were noted as roosting above the plastic sheet at the time of collection. Suggestive of a mixed under-roost sample from *P. poliocephalus* *P. alecto*.
ARCLU010_26_1	TACTTCAACTACAAGAACCACAATGACAAACATCCGTAAATCACACCCAC<br>TATTCAAAATTATCAACGACTCACTGATCGACCTACCCGCCCCATCAAGT<br>ATTTCCTCATGATGAAACTTCGGCTCACTACTAGGCATCTGTCTAGCCAT<br>CCAAATCCTAACAGGACTGTTCCTAGCCATACACTACACTTCAGACACAA<br>CGACCGCCTTCCAATCCGTAACTCATATCTGCCGAGACGTAAATTACGGA<br>TGAATCCTACGTTATTTGCATGCTAACGGAGCATCCATATTCTTCATCTG<br>CCTATTCTTACATGTAGGCCGAGGCCTCTACTATGGATCTTACATTTACA<br>AAGAAACCTGAAACGTAGGTGTTATTCTCCTATTCGCCGTAATAGCAACA<br>GCC	78.4	*P. alecto*<br>Cytb sequence has 98.16% match to *P. griseus* (KJ532423.1) and 95.78% match to *P. alecto* (KF726143.1). The sequence contained multiple mixed peaks, where the alternative peak is consistent with *P. alecto* sequences.<br>Two *P. alecto* were noted as roosting above the plastic sheet at the time of collection. As described above for ACCLU004_22_1F, *P. alecto* is poorly resolved relative to other species, and include lineages closely related to *P. griseus*. Suggestive of a mixed under-roost sample from more than one *P. alecto*.
ARLIS002_55_1	TATTTCAACTACAAGAACACCAATGACCCCAATACGCAAAACTAACCCCC	94.0	*Homo sapiens*

Sample ID	Cytochrome b sequence	HQ, %*	Assessment
	TAATAAAATTAATTAACCACTCATTCATCGACCTCCCCACCCCATCCAAC<br>ATCTCCGCATGATGAAACTTCGGCTCACTCCTTGGCGCCTGCCTGATCCT<br>CCAAATCACCACAGGACTATTCCTAGCCATGCACTACTCACCAGACGCCT<br>CAACCGCCTTTTCATCAATCGCCCACATCACTCGAGACGTAAATTATGGC<br>TGAATCATCCGCTACCTTCACGCCAATGGCGCCTCAATATTCTTTATCTG<br>CCTCTTCCTACACATCGGGCGAGGCCTATATTACGGATCATTTCTCTACT<br>CAGAAACCTGAAACATCGGCATTATCCTCCTGCTTGCAACTATAGCAACA<br>GCC		Cytb sequence has 100% match to *Homo sapiens*, indicating the sample was likely contaminated.
ARNAM005_2_1	TACTTCAACTACAAGAACCACAATGACAAACATCCGTAAATCACACCCAC<br>TATTCAAAATTATCAACGACTCACTGATCGACCTACCCGCCCCATCAAGT<br>ATTTCCTCATGATGAAACTTCGGCTCACTACTAGGCATCTGTCTAGCCAT<br>CCAGATCCTAACAGGACTGTTCCTAGCCATACACTACACTTCAGACACAA<br>CGACCGCCTTCCAATCCGTAACTCATATCTGCCGAGACGTAAATTACGGA<br>TGAATCCTACGTTATTTGCATGCTAACGGAGCATCCATATTCTTCATCTG<br>CCTATTCCTACATGTAGGCCGAGGCCTCTACTATGGATCTTACATTTACA<br>AAGAAACCTGAAACGTAGGTGTTATTCTCCTATTCGCCGTAATAGCAACA<br>GCC	95.7	*P. alecto*<br>Cytb sequence has 98.51% match to *P. alecto* (KF726143.1).<br>Although two *P. poliocephalus* were noted as roosting above the plastic sheet at the time of collection, sequence data is suggestive of a sample from *P. alecto*.
ARNAM005_12_1	TACTTCAACTACAAGAACCACAATGACAAACATCCGCAAATCACACCCAC<br>TATTCAAAATTATCAACGACTCACTGATCGACCTACCCGCCCCATCAAGT<br>ATTTCCTCATGATGAAACTTCGGCTCACTACTAGGCATCTGCCTAGCCAT<br>CCAAATCCTAACAGGACTATTCCTAGCCATACACTACACTTCAGACACAA<br>CGACCGCCTTCCAATCCGTGACCCATATCTGCCGAGACGTAAATTACGGA<br>TGAATTCTGCGTTATTTACATGCTAACGGAGCATCCATATTCTTCATCTG<br>CCTATTCTTACATGTAGGCCGAGGCCTCTACTACGGATCTTACATCTACA<br>AAGAAACCTGAAACGTAGGTGTTATTCTCCTATTTGCCGTAATAGCAACA<br>GCC	55.6	*P. alecto*<br>Cytb sequence has 98.51% match to *P. alecto* (KF726143.1). Low-quality sequence includes non-diagnostic mixed peaks.<br>Four *P. alecto* were noted as roosting above the plastic sheet at the time of collection.
ARSUN015_15_1	TACTTCAACTACAAGAACCACAATGACAAACATCCGAAAATCACACCCAC<br>TATTCAAAATTATCAACGACTCACTAGTCGACCTACCCGCTCCATCAAGT<br>ATCTCATCATGATGAAACTTTGGCTCACTACTAGGCATCTGCCTAGCCAT<br>CCAAATCCTGACAGGACTGTTCCTAGCCATACACTACACTTCAGACACAA<br>CAACCGCCTTCCAATCCGTGACTCACATCTGCCGAGACGTAAACTACGGA<br>TGAATCCTCCGCTACTTACACGCTAACGGAGCATCCATATTCTTCATCTG<br>CCTATTCCTGCATGTGGGCCGAGGCCTCTACTACGGATCTTACATCTATA<br>AAGAGACCTGAAACGTAGGTGTCATCCTTCTATTTGCCGTAATAGCAACA<br>GCC	96.2	*P. poliocephalus*<br>Cytb sequence has 99.48% match to *P. poliocephalus* (KJ532404.1). Four *P. poliocephalus* were noted as roosting above the plastic sheet at the time of collection
ARDOR001_S2_1	TACTTCAACTACAAGAACCACAATGACAAACATCCGCAAATCACACCCAC<br>TATTCAAAATTATCAACGACTCACTGATCGACCTACCCGCCCCATCAAGT<br>ATTTCCTCATGATGAAACTTCGGCTCACTACTAGGCATCTGCCTAGCCAT<br>CCAAATCCTAACAGGACTATTCCTAGCTATACACTACACTTCAGACACAA<br>CGACCGCCTTCCAATCCGTGACCCATATCTGCCGAGACGTAAATTACGGA<br>TGAATTCTGCGTTATTTACATGCTAACGGAGCATCCATATTCTTCATCTG<br>CCTATTCTTACATGTAGGCCGAGGCCTCTACTACGGATCTTATATCTACA<br>AAGAAACCTGAAACGTAGGTGTTATTCTCCTATTTGCCGTAATAGCAACA<br>GCC	96.1	*P. alecto*<br>Cytb sequence has 100% match to *P. alecto* (KJ532406.1) and *P. conspicillatus* (KJ532443.1).<br>*P. conspicillatus* are not present in NSW and *P. alecto* were noted at the roost at the time of collection. Sequence data is suggestive of a sample from P. alecto.

*The percentage of bases in a sequence that are high quality (HQ) in the sequence.
